# Telepharmacy Consultations (TPCs) in Local Pharmacies—A Bi-Centric Survey of Customer Opinions

**DOI:** 10.3390/pharmacy13060177

**Published:** 2025-12-08

**Authors:** Nathalie Floch, Philipp Harand, Chris Graichen, Thilo Bertsche

**Affiliations:** 1 Clinical Pharmacy Department, Institute of Pharmacy, Medical Faculty, Leipzig University, 04103 Leipzig, Germany; 2 Drug Safety Center, Leipzig University and Leipzig University Hospital, 04103 Leipzig, Germany; 3 City Pharmacy, 66111 Saarbrücken, Germany; 4 Pharmacy at Saint George, 04158 Leipzig, Germany

**Keywords:** remote consultation, video conferencing, community pharmacy services, surveys and questionnaires, self-assessment

## Abstract

*Background*: Telepharmacy consultations (TPCs) became a routine element of pharmacy operations. However, there is limited data available on local pharmacy customer feedback related to TPC. *Methods*: A customer survey was developed seeking feedback on TPC. The pharmacy customers were invited to complete the survey in two local pharmacies in Germany. The survey and corresponding informed consent form were approved by the Ethics Committee. *Results*: In total, 178 pharmacy customers were enrolled (median age 41–50 years). From those, 37% agreed when asked whether they were generally interested in TPC. A total of 37% had the nearest pharmacy 5–15 min from their home. A total of 42% visited their pharmacy quarterly. A total of 36% used technical devices in median 1–2 h per days. A total of 33% classified their own digital skills at least as sufficient. A total of 59% would use their smartphone as a potential device for TPC. A total of 83% rated it as (slightly) important that the pharmacist providing TPC can be heard clearly. A total of 76% each (strongly) agreed that an argument for TPC would include limited mobility or pandemic/quarantine. A total of 33% (strongly) agreed that a key argument against TPC were technical requirements. A total of 75% considered situations of immobility to be the most important future perspective for TPC. *Conclusions*: Many pharmacy customers see TPC as an opportunity, e.g., in cases of limited mobility or during pandemic or quarantine. However, the use of appropriate technology can be a limiting factor.

## 1. Introduction

Around the world, local pharmacies play a significant role in the provision and dispensing of medicinal products and medical devices [1]. Furthermore, public health measures are implemented to promote health and prevent disease [2]. Local pharmacies are increasingly recognized and utilized as first contact points for a wide range of health complaints, particularly minor ailments and preventive services in the area of self-care and self-medication [3]. Local pharmacies are also responsible for checking the safety of medical prescriptions and advising patients for prescribed medicines [4]. The advice is critical as without appropriate advice, medicines are often not intuitive enough to be administered correctly and can therefore be ineffective and risky [5,6,7].

This shows that the tasks of a pharmacy go far beyond purely logistical requirements.

However, the question arises as to how these can be performed if there is no local pharmacy or if the nearest one is a long way away. What if, for example, customers are immobile or contact is restricted for epidemiological reasons?

Since the Coronavirus disease 2019 (COVID-19) pandemic, the frequency of using virtual meetings, video consultations and remote working has been increasing—it has even made many people aware of them for the first time [8]. Telepharmacy consultations (TPCs) are defined as the provision of pharmaceutical care by pharmacies using telecommunication technologies for patients or customers who are located at a distance [9]. According to a recent review, TPC could replace or supplement pharmaceutical services, thereby promoting future innovations in healthcare [9]. TPC might offer many potential advantages: examples include easy access to healthcare services even in rural areas, economic benefits, increased customer satisfaction (e.g., no travel is required), and effective patient counseling [10]. And all of this is possible even when there is a shortage of local pharmacists and pharmacy services from local pharmacies. However, there are also issues that can make practical implementation of TPC difficult. Examples include requirements for hardware, software, connectivity, and operational costs [10]. The institutionalized pharmacists in Germany support TPC and the care options that TPC services offer patients. According to the Federal Union of German Associations of Pharmacists (ABDA), TPC should be offered by local pharmacies in appropriate cases [11]. It is seen as an additional tool in the complex process of providing medication. The ABDA sees the limitations of TPC in Germany in the fact that only pharmacists should offer it. In addition, it should be aimed at people who are personally known to the pharmacy or who have prepared for contact with the pharmacy. At the 2022 general meeting of the German Pharmacists’ Day, it was decided to further develop TPC in the interests of patients.

TPC outside of pharmacies, without pharmacists, or only between pharmacies is not referred to as TPC. According to the ABDA, TPC refers exclusively to the pharmacy–patient relationship. Pharmacies in Germany already offer several TPC services. However, these still have potential for further development in order to bring the expertise of pharmacists more strongly into the care process [11].

However, the extent to which local pharmacy customers would actually take up TPC offers from their local pharmacy is still unknown. Another question where only limited data is available [12] is whether customers have sufficient technical knowledge to use remote applications in addition to the availability of suitable devices and internet access. The availability of high-speed internet access, facilitating a good quality connection for counseling, can be very limited in rural areas [13]. However, TPC appears to be particularly useful in those rural areas [14], where, in contrast to large cities [15], the nearest pharmacy is far away [16]. Therefore, especially in larger and medium cities, the question of whether customers and potential users would really like to receive TPC from their pharmacy arises.

Although there is some data in the literature on TPC around the world [17], the question is whether this information can be transferred between countries. This is not only because of differences in population density and structure. The pharmacy system in Germany [18] is in some respects completely different from that in many other countries. In addition, new opportunities for patient participation in telemedicine [19] and changes in internet use patterns among older adults [20] also require an updated assessment, as recently published data, e.g., before the COVID-19 pandemic, are of limited relevance to the current situation and therefore require additional investigation.

The study was designed as a survey, investigating customer feedback on TPC. Two pharmacies were selected in different cities in East and West Germany to complete a survey on TPC designed for purpose of this study.

## 2. Materials and Methods

*Participants and setting*: Two local pharmacies in two large German cities, i.e., in Leipzig (619,879 inhabitants on 31 December 2023, located in Eastern Germany) and in Saarbrücken (186,283 inhabitants on 31 December 2023, located in Western Germany) invited all customers to participate in a questionnaire-based survey (Supplementary Materials) during the periods of 2 January 2021—15 March 2021 (Leipzig) and 2 August 2021—31 December 2021 (Saarbrücken). Leipzig was chosen based on the location of our institute (a city in East Germany). Saarbrücken was chosen as a city in West Germany as a suitable counterpart. The pharmacy was part of a cooperative network with which our department frequently carried out projects (Leipzig) and the pharmacy where a doctoral student (N.F.) worked (Saarbrücken). TPC as a supplement to face-to-face service has only recently become possible in principle in the participating pharmacies, but was not yet being used to any great extent at the time of the survey.

*Ethical vote*: The Ethics Committee of the Medical Faculty of Leipzig University has approved the ethics application including the study protocol, customer information, and the questionnaire as the basis of the survey. The positive ethics vote (No. 515/20-ek) was issued on 17 November 2020. All participating customers gave their written informed consent in advance. The cooperating pharmacy owners were informed in advance and also agreed to the study.

*Study design*: Enrolled pharmacy customers were asked questions in a newly developed questionnaire in a prospective descriptive non-interventional bi-centric study design.

*Inclusion criteria*: As inclusion criteria, customers had to be of legal age (at least 18 years old) and to have full legal capacity. Additionally, they had to have sufficient language skills and had to have the physical and mental ability to participate in a written survey. In addition, their written consent to answer the questionnaire and to use the anonymized data for scientific purposes was required in advance. In this context, all customers were invited regardless of whether they were regular customers or not, and regardless of whether they were filling in a prescription, requesting self-medication or buying a product or not. The questionnaire was accessed online without storing email addresses or IP addresses.

*Study protocol*:

1. A questionnaire to be used within this study was designed by an expert panel, and was pretested and piloted by participants independent of the main study.
2. Two cooperating pharmacies located in East (Leipzig) and West (Saarbrücken) Germany were invited to participate in the survey.
3. Pharmacy customers fulfilling the inclusion criteria were invited to participate by the pharmaceutical staff in the participating pharmacies during the study period. Either a paper questionnaire was provided directly at the customer’s request, or a flyer with an online link was provided as an online-based questionnaire to be completed later. Enrolled Customers were invited to fill in the questionnaire (designed for purpose of this study) in an online- or a paper-based version based on personal preference.

*Questionnaire*: The questionnaire was designed by an expert panel of experienced pharmacists (the authors of this study). All authors were well-experienced in the setting of community pharmacies with several years of experience. The last author spent many years developing and updating electronic knowledge systems. This was performed in close collaboration with medical informatic specialists. The web-based program SoSci Survey (Version of 2021, SoSci Survey GmbH, 81929 München, Germany) was used as a tool for development of the questionnaire.

As the survey was aimed at a wide audience of pharmacy customers, the answers were deliberately kept simple. This was a judgment of the panel of experts and a result of the pretest. One way of performing this was to give answers on categories or intervals where possible. The option to enter individual numbers or text would have been desirable for the analysis but would create a greater challenge for participants to complete the questionnaire than ticking comparably simple response options. The pharmacist administering the survey was allowed to assist by reading aloud without comments. Via access links, as a QR code or hyperlink, respondents were forwarded to the respective questionnaires and completed them in digital form. The results of the complete questionnaires were saved in SoSci Survey and could be downloaded for analysis in Microsoft Excel or SPSS. SoSci Survey is free of charge for surveys conducted as part of academic research. Within this survey, participants could also submit a paper version if they wished. In this case, the data was added manually.

*Quality assurance of the survey*: The questionnaire was pretested [21] with the following methods available for conducting a cognitive pretest: paraphrasing, sorting, probing and thinking aloud. The pretest was performed in interviews of 21 independent participants, including 13 without a pharmaceutical background. In the pretest phase, two additional open questions were added (further advantages and further disadvantages). Questions about working in a healthcare profession were added. The question about the assessment of one’s own therapy quality was canceled, as it proved difficult for many participants to evaluate. The questions were discussed with the expert panel for their relevance and only then were they retained or discarded. The scale display was standardized and the order of the questions was changed several times as a result of the pretests. A pilot study was carried out as a final evaluation of the questionnaire under real conditions in a local pharmacy. No further problems or comments were expressed by the testing group.

The study was monitored by two pharmacists (N.F. and P.H.) from the expert panel after detailed internal coordination and all the pharmaceutical staff involved in the pharmacies were trained in advance.

*Statistics and data evaluation*: Customer’s answers to the questions were documented in a template at the online question tool ScoSci-Survey. All data are given descriptively in absolute and relative values. Data received from the survey were analyzed using SoSci Survey and were downloaded as an electronic documentation file in Microsoft Excel (for Windows, Microsoft Corporation, Redmond, WA, USA). Two datasets were compared between the two settings via SPSS (for Windows, Version 29, IBM SPSS Statistics) using the chi-square test (for dichotomous data in the two independent customer groups) or the Mann–Whitney U test (for ordinal scaled data in the two independent customer groups), as appropriate.

## 3. Results

### 3.1. Participant Characteristics

A total of 178 (80 female, 66 male, 2 diverse, 30 not specified) customers of participating pharmacies gave their written informed consent, from those 113 in Leipzig and 65 in Saarbrücken. The median age group was 41 to 50 years. A total of 78% (139/178) had a master, magister, or state examination as a professional qualification. Details of participant characteristics are presented in Table 1.

### 3.2. Pharmacy and Drug Supply

Overall, 37% (66/178) agreed when asked whether they were generally interested in TPC. This number was 38% (43/113) in Leipzig and 37% (24/65) in Saarbrücken. No significant differences between the two sites were found. A total of 37% (66/178) participating customers reported to have the nearest pharmacy 5–15 min from their home. A total of 42% (74/178) visited their pharmacy quarterly, 48% (86/178) had 0–1 prescribed medications and 51% (91/178) 0–1 self-medications. Details of customer reports on their pharmacy and drug supply are presented in Table 2.

### 3.3. Technical Performance

A total of 36% (64/178) participating customers reported using technical devices in median 1–2 h per day for private use. A total of 33% (59/178) classified their own digital skills at least as sufficient. A total of 59% (105/178) would use their smartphone as a potential device for TPC. Details of customer reports on their technical performance are presented in Table 3.

### 3.4. Preconditions for TPC

**Figure 1:**
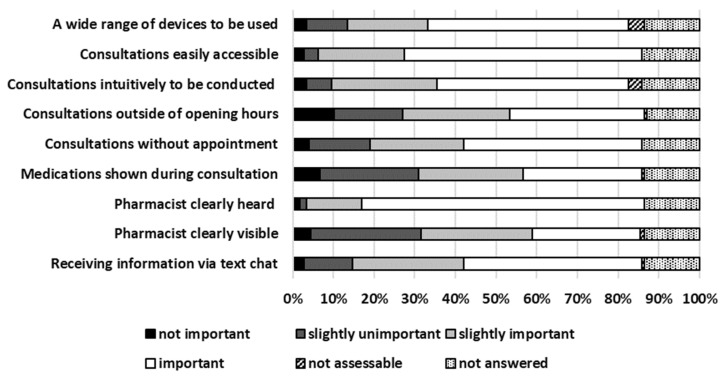
Preconditions for TPC.

Out of all participating customers, the top five preconditions considered very important or important for TPC were “pharmacist heard clearly”, “consultations easily accessible”, “a wide range of devices to be used”, “consultations intuitively to be conducted”, and “receiving information via text chat”. Details about preconditions for TPC are presented in Figure 1.

### 3.5. Arguments for TPC

**Figure 2:**
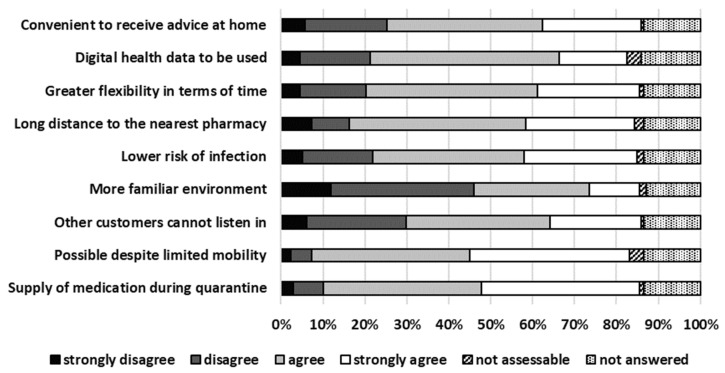
Arguments for TPC.

The five most strongly agreed and agreed arguments for TPC were “possible despite limited mobility”, “supply of medication during quarantine”, “long distance to the nearest pharmacy”, “greater flexibility in terms of time”, and “lower risk of infection”. Details about arguments for TPC are presented in Figure 2.

### 3.6. Arguments Against TPC

**Figure 3:**
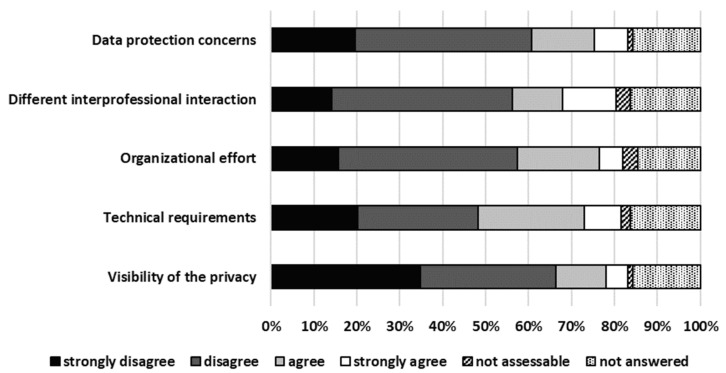
Arguments against TPC.

The technical requirements needed for TPC such as telephone, computer, and high-speed internet access were the top priority in the arguments against TPC. The arguments against TPC are presented in Figure 3.

### 3.7. Future Perspectives for TPC

**Figure 4:**
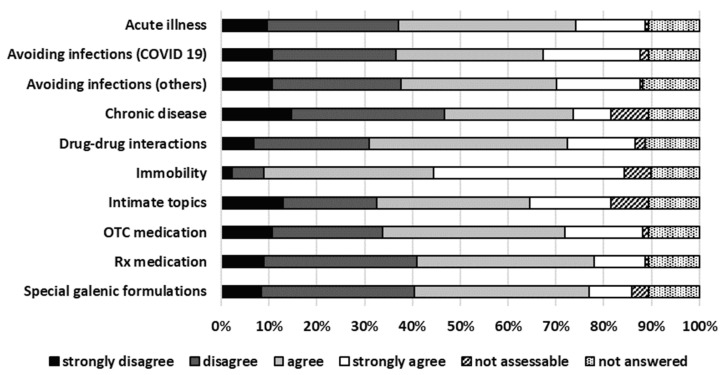
Future perspectives for TPC.

A total of 71% (126/178) participants strongly agreed and agreed that TPC is a good addition to on-site consulting. Details about future perspectives are presented in Figure 4.

## 4. Discussion

### 4.1. General Considerations

Modern pharmacy demands new tools to provide optimal service. Consultations should keep up to speed with increase in online retailing. TPC has developed into a tool that is frequently used both privately and professionally in many areas. So, what should stand in the way of using such offers for customers of local pharmacies as well? What is the argument against receiving a logistical and advisory support from a trusted contact person at the local pharmacy, using remote technology? A key to success of TPC lies in customer’s willingness to engage in TPC and ability to use remote technology.

### 4.2. Most Important Findings

The prospective bi-centric survey found that more than a third of respondents agreed when asked if they were generally interested in TPC. Participants were predominantly middle-aged, many used telecommunication equipment and about a third rated their knowledge of this equipment as at least sufficient. The smartphone was named as preferred device for TPC to their pharmacy. If a consulting pharmacist can be heard clearly on the device, the customers are also open to TPC. Customers see the benefits of TPC, particularly in cases where mobility is limited, for example, due to quarantine or illness. The participants believe that consultations can have limited effect since the technology poses a challenge and since there are no personal interactions with the pharmacist. The majority of the respondents who were also a customer of a local pharmacy will in future see the TPC option as a supplement rather than a replacement for personal advice in their pharmacy. The survey results aim to draw conclusions about the extent to which local pharmacies should be prepared for TPC in the future.

### 4.3. Methodological and Technical Aspects

The survey periods differ considerably between the two sites: Leipzig (January–March 2021) and Saarbrücken (August–December 2021). This temporal gap, and potential seasonal or epidemiological differences (related to the COVID-19 pandemic), may have influenced participants’ attitudes. However, no significant differences were found between the two settings. In conclusion, the time or the setting of the assessment did not influence the results and there is no occurring bias factor. Furthermore, only pharmacy clients who may be more receptive to pharmaceutical counseling were included in the study. This study aimed to know the opinions of people who are actual customers of pharmacies in order to improve customer satisfaction, not to attract new customers. Of course, it will also be interesting to survey other groups outside of pharmacies in the future.

Older people today are not necessarily unfamiliar with remote services, partly because of the COVID-19 pandemic. The use of devices such as smartphones is increasing significantly, simply because generations have now reached an older age that is already familiar with these technical communications such as remote services during their working lives. In any case, respondents themselves often stated that they had the technical skills and consider them crucial to the success of remote services.

### 4.4. Comparison to Literature

While the topic of telemedicine and remote conferencing considering medicine and physicians is discussed frequently in many settings including aspects, e.g., from the COVID-19 pandemic to nursing homes to pain management [22,23,24,25], there is comparatively little literature on corresponding topics in relation to pharmacies. As reported in a review [26], pharmacy telemedicine interventions in the outpatient or ambulatory setting have an overall positive impact on outcomes related to clinical disease management, patient self-management, and adherence in the management of chronic diseases. Most of the projects included in the review, were primarily conducted via telephone. Commonalities among studies with positive impact included utilization of continuous or scheduled models via telephone, with frequent monitoring and interventions [26]. In line with this report, the study also found the smartphone as the preferred device for TPC. Even if TPC is conceivable, a simple telephone call is also possible. A particularly interesting concept has been presented in [27]: In assisted teleconsultation, a telemedical intervention was carried out with a patient in the presence of a healthcare professional on one side and an expert on the other. The authors conclude that a young population with semi-urgent medical problems could be managed in the pharmacy using assisted teleconsultation with a primary care physician. In contrast, our survey focused on a relatively simple TPC, where the patient (usually from home) speaks to a pharmacist without having to visit the pharmacy. Specific medication requests could then be delivered in a short time using a delivery service. Of course, the concept could also be extended towards TPC with the involvement of external experts, including physicians.

A distinction should be made, in this regard, between our method, in which telecommunication is established from the patient to the healthcare professional, and telemedicine/telepharmacy services, as reported in [28]. In the latter, the focus is on involving specialists by TPC in local counseling processes and thus making those specialists available in areas where they are not available onsite or only available to a limited extent. Interestingly, a review [29] analyzing telemedical consultations comes to a very similar conclusion as performed in this study: teleconsultations can reduce the number of visits to the physician, especially in lockdown situations, and both patients and physician are satisfied with the method. Nevertheless, like our study with participating pharmacy customers, the authors conclude that a physical consultation cannot be completely replaced by a TPC due to its limitations.

Our study should be compared to another study [30] in which groups of people opted for a privately funded service that offered physician’s appointments via video conferencing in pharmacies. Reasons for using the service included a shorter waiting time as compared to a routine appointment or appointment at a more convenient time.

A focus on patients with chronic conditions who require long-term medication is reported in [31]. A telepharmacy service can be of benefit to this particular group of patients, which are particularly vulnerable to infections, especially in quarantine situations. Out of 39 patients in [31], 82% were young or middle-aged. The problems of long-term medication for patients with chronic diseases were effectively addressed according to the authors. This is consistent with the experience of this study that, in principle, younger and tech-savvy patients or pharmacy customers are more likely to participate in telepharmacy services or to be interested in a survey on the subject. 

Of particular interest is the question of what impact the COVID-19 pandemic had specifically on TPC services. This was examined in an article [32]: A total of 19 relevant original research articles were evaluated. According to these studies, telepharmacy was used to remotely review and optimize medication, assess medication adherence, administer and deliver medication, educate and advise patients, promote disease prevention, collaborate with healthcare providers, and monitor treatment outcomes. However, despite an overall positive conclusion, the authors note that randomized clinical trials are needed to investigate the long-term efficacy and cost-effectiveness of telepharmacy services [32]. 

The authors in [9] come to an even more optimistic conclusion on the same topic: Telepharmacy could potentially replace or supplement pharmaceutical activities, thereby facilitating future innovation in the healthcare sector [9]. Of particular interest is how the COVID-19 pandemic has led to changes in services or their effectiveness. To this end, the authors in a very current publication [33] examined in 40 finally included studies whether compared with no telepharmacy services or usual care (i.e., face-to-face pharmaceutical services), telepharmacy services probably increased patient medication adherence, and may reduce the occurrence of adverse events and improve the proportion of patients who were satisfied with medication. However, as the authors concluded [33], the effectiveness of telepharmacy services was not different before and after the outbreak of the COVID-19 pandemic except for medication adherence, which also increased over time.

### 4.5. Limitations

When drawing conclusions and generalizing the results of this survey, the following limitations were considered: Firstly, this survey was performed in the context of the COVID-19 pandemic. It remains to be seen to what extent both positive (e.g., improved technical possibilities) as well as negative experiences (e.g., lack of personal human contact) from this period would equilibrate or continue in the future. Secondly, it should be remembered that the study surveyed pharmacy customers. The results do not allow us to draw conclusions about the extent to which people who do not visit local pharmacies at all and prefer to buy their medicines online have a different attitude towards TPC. They may consider them more positive or do not consider consultations necessary at all. Thirdly, it should be remembered that although two different locations were included in the study, and pharmacies and the results were largely very similar, extrapolations to pharmacies, in general, should only be made with caution.

## 5. Conclusions

More than a third of pharmacy customers are generally interested in TPC regarding pharmaceutical advice. This typical pharmacy customer likes to use the smartphone, which she or he also uses privately as the most important potential device. She or he considers her or his technical skills to be at least sufficient. For consultation to be successful, the pharmacist should be easy to understand. A TPC makes sense particularly in case of limited mobility due to quarantine or illness. For this reason, TPC will continue to be seen as a complementary offer to on-site consultations in pharmacies. This is because of the necessary technical requirements, but also the poorer interaction than with face-to-face communication in the pharmacy, which are perceived as negative.

## Figures and Tables

**Table 1:** Participant characteristics.

Item	Leipzig (*N* = 113)	Saarbrücken (*N* = 65)	Total (*N* = 178)
**Gender (f/m/d/not specified)**	53/47/1/12	27/19/1/18	80/66/2/30
**Age [median: 41 to 50 years]**			
<21	7	1	8
21–30	9	8	17
31–40	28	11	39
41–50	13	7	20
51–60	19	11	30
61–70	16	2	18
71–80	5	5	10
>80	4	2	6
Not specified	12	18	30
**Highest level of formal educational qualification (multiple answers possible)**			
(So far) without a professional qualification	7	1	8
Apprenticeship	43	9	52
Bachelor	60	38	98
Master, magister, state examination	95	44	139
Diploma	8	3	11
PhD, MD, Dr (or equivalent)	90	45	135
Another professional qualification	13	2	15

**Table 2:** Pharmacy and drug supply.

Item	Category	Leipzig (*N* = 113)	Saarbücken (*N* = 65)	Total (*N* = 178)
**Distance (travel time) to most frequently visited pharmacy [%] (median: 5 to 15 min)**				
	<5 min	35	13	48
	5–15 min	49	17	66
	16–30 min	9	16	25
	>30 min	7	2	9
	Not specified *	13	17	30
**Frequency of pharmacy visit (median: quarterly)**				
	Daily	0	0	0
	Weekly	13	7	20
	Monthly	26	14	40
	Quarterly	55	19	74
	Yearly	7	6	13
	Less than yearly	0	2	2
	Not specified *	12	17	29
**Number of quarterly prescribed medication (Rx drugs) (median: 0–1)**				
	0–1	56	30	86
	2–3	21	9	30
	4–5	15	4	19
	>5	9	4	13
	Not specified *	12	18	30
**Number of quarterly bought self-medication (OTC drugs) (median: 0–1)**				
	0–1	66	25	91
	2–3	28	16	44
	4–5	5	5	10
	>5	2	1	3
	Not specified *	12	18	30

Not specified *: * Includes missing responses (due to premature termination, skipping the question) and the deliberate selection of “I cannot judge.”

**Table 3:** Technical performance.

Item	Category	Leipzig (*N* = 113)	Saarbrücken (*N* = 65)	Total (*N* = 178)
**Private, daily usage time of technical devices (median: 1–2 h)**				
	<1 h	18	14	32
	1–2 h	44	20	64
	3–4 h	24	9	33
	5–6 h	5	2	7
	>6 h	8	2	10
	“I do not spend any time”	2	1	3
	Not specified *	12	17	29
**Self-assessment of digital skills (based on German school grade system) (median: satisfactory)**				
	Deficient	4	0	4
	Poor/inadequate	4	3	7
	Sufficient	8	1	9
	Satisfactory	14	6	20
	Good	10	7	17
	Very good	9	4	13
	I cannot judge	3	0	3
	Not specified *	61	44	105
**Devices potentially used for TPC**				
	Smartphone	61	44	105
	Tablet	37	22	59
	Laptop/notebook/computer	64	28	92
	Other devices	0	0	0
	More than one device	51	30	81

Not specified *: * Includes missing responses (due to premature termination, skipping the question) and the deliberate selection of “I cannot judge.”

## Data Availability

The data presented in this study are available on request from the corresponding author, insofar as this is justifiable for ethical and data protection reasons.

## Supplementary Materials

The following supporting information can be downloaded at https://www.mdpi.com/article/10.3390/pharmacy13060177/s1, Supplement S1: Questionnaire.

## Abbreviations

Coronavirus disease 2019 (COVID-19), Federal Union of German Associations of Pharmacists (ABDA), Over-the-Counter (OTC), Telepharmacy Consultation (TPC).
