# Supplementary material for: Telepharmacy Consultations (TPCs) in Local Pharmacies—A Bi-Centric Survey of Customer Opinions
Source: Pharmacy (Basel). 2025 Dec 8;13(6):177. doi: 10.3390/pharmacy13060177 (PMC12736953; doi:10.3390/pharmacy13060177)
Supplement: Supplementary file 1 [file pharmacy-13-00177-s001.zip › pharmacy-3954438-supplementary.pdf]

Supplement S1: Questionnaire

Dear Sir or Madam,

Thank you for your willingness to participate in the survey on the topic of "Remote consultations by local community pharmacies."

For a remote consultation, you can schedule an appointment with your pharmacy and will then receive a link via email that will give you access to a consultation with a pharmacist or PTA. The pharmaceutical staff can then advise you online with image (camera) and sound, via computer, smartphone, or tablet, about medications and answer questions about your health.

The following questionnaire will ask you questions about your personal attitude toward remote consultations, as well as your concerns and wishes in this regard. The goal of remote consultations is to ensure comprehensive and optimal supply of medicines. Even if you have never participated in a remote consultation before, your opinion is very valuable to us in developing a remote consultation.

The process takes **approximately 15 minutes**.

This survey is a collaborative project between the Pharmacy at Sanct Georg and the Clinical Pharmacy at Leipzig University. By voluntarily participating, you consent to the storage of the data collected here. All data will be treated confidentially and anonymously. Anonymous means that no conclusions can be drawn about your identity. Participation is not compensated.

If you have any questions, members of the Pharmacy at Sanct Georg (Philipp Harand, Chris Graichen) and the Clinical Pharmacy (Nathalie Szafarczyk) are available during pharmacy opening hours at +49 341 900 400 or by email at any time (nathalie.szafarczyk@uni-leipzig.de, philipp.harand@studserv.uni-leipzig.de, c.graichen@georg-apotheke-leipzig.de).

1. Before participating in this survey, had you heard or read that a local pharmacy offers remote consultations?
If you answer “No” to this question, please continue with question 3.
☐ No, I have not heard of remote consultations.
☐ Yes, I have heard of remote consultations, but have not yet used them.
☐ Yes, I have already heard of remote consultations and have already used them.

2. How did you find out about remote consultations offered by a local pharmacy?
Multiple selections possible
☐ Advertising (e.g., print media)
☐ Family, friends, acquaintances
☐ Physician
☐ Pharmacy
☐ Health insurance company
☐ Internet, if so, where:
☐ Other (please specify):

3. Imagine your pharmacy offers remote consultations as an alternative to in-person consultations. How interested are you in using remote consultations with your local pharmacy?
☐ very small
☐ small
☐ large
☐ very large
☐ I cannot assess

4. How interested are you in using a remote consultation in the following situations?
Other situations
(please state):

If I have an acute illness (e.g., flu, cold, fever).
☐ very small
☐ small
☐ large
☐ very large
☐ I cannot assess

If I have a chronic illness (e.g., high blood pressure, diabetes).
☐ very small
☐ small
☐ large
☐ very large
☐ I cannot assess

If I would like advice on medications available without a prescription from a pharmacy.
☐ very small
☐ small
☐ large
☐ very large
☐ I cannot assess

If I have questions about medications my doctor has prescribed.
☐ very small
☐ small
☐ large
☐ very large
☐ I cannot assess

If I would like advice on intimate topics (e.g., athlete's foot, hemorrhoids, vaginal yeast infection).
☐ very small
☐ small
☐ large
☐ very large
☐ I cannot assess

If I would like to have the use of special medications explained (e.g., eye drops, suppositories, dry syrups).
☐ very small
☐ small
☐ large
☐ very large
☐ I cannot assess

To receive advice on possible interactions with my medications.
☐ very small
☐ small
☐ large
☐ very large
☐ I cannot assess

To avoid infections during the coronavirus pandemic.
☐ very small
☐ small
☐ large
☐ very large
☐ I cannot assess

To avoid infection (e.g., during flu season).
☐ very small
☐ small
☐ large
☐ very large
☐ I cannot assess

If I'm not mobile and want to contact a pharmacy (e.g., broken leg, bedridden).
☐ very small
☐ small
☐ large
☐ very large
☐ I cannot assess

☐ Other situations (please state):

5. How important are the following aspects of a remote consultation to you?
That a remote consultation is easily accessible.
☐ unimportant
☐ rather unimportant
☐ rather important
☐ important
☐ I cannot assess

That a remote consultation is intuitive.
☐ unimportant
☐ rather unimportant
☐ rather important
☐ important
☐ I cannot assess

That a remote consultation can be conducted without prior appointment.
☐ unimportant
☐ rather unimportant
☐ rather important
☐ important
☐ I cannot assess

That I can participate in a remote consultation using as many different devices as possible (e.g., smartphone, tablet, computer).
☐ unimportant
☐ rather unimportant
☐ rather important
☐ important
☐ I cannot assess

That I can receive advice outside of opening hours.
☐ unimportant
☐ rather unimportant
☐ rather important
☐ important
☐ I cannot assess

That the consultant is clearly visible.
☐ unimportant
☐ rather unimportant
☐ rather important
☐ important
☐ I cannot assess

That the consultant is clearly understandable.
☐ unimportant
☐ rather unimportant
☐ rather important
☐ important
☐ I cannot assess

That the medication I am being advised on can be seen.
☐ unimportant
☐ rather unimportant
☐ rather important
☐ important
☐ I cannot assess

That I receive information via text chat (e.g., on the correct intake and dosage of my medication).
☐ unimportant
☐ rather unimportant
☐ rather important
☐ important
☐ I cannot assess

☐ Other reasons (please state):

6. In your opinion, to what extent do the following reasons speak against remote consultations?
The organizational effort (e.g., scheduling an appointment).
☐ not at all
☐ a little
☐ strong
☐ very strong
☐ I cannot assess

The technical requirements (e.g., required stable internet, adequate picture and sound quality).
☐ not at all
☐ a little
☐ strong
☐ very strong
☐ I cannot assess

It's a different kind of interpersonal interaction compared to a face-to-face conversation (e.g., facial expressions and gestures).
☐ not at all
☐ a little
☐ strong
☐ very strong
☐ I cannot assess

The insight into my privacy (e.g., visibility of my living environment in the background).
☐ not at all
☐ a little
☐ strong
☐ very strong
☐ I cannot assess

Possible data protection concerns.
☐ not at all
☐ a little
☐ strong
☐ very strong
☐ I cannot assess

☐ Other reasons (please state):

7. In your opinion, to what extent do the following reasons speak in favor of remote consultations?

Greater flexibility in my daily routine (e.g., no waiting at the pharmacy).
☐ not at all
☐ a little
☐ strong
☐ very strong
☐ I cannot assess

It's more convenient for me, as it takes place from home.
☐ not at all
☐ a little
☐ strong
☐ very strong
☐ I cannot assess

The risk of infecting myself or others is reduced (e.g., during flu season).
☐ not at all
☐ a little
☐ strong
☐ very strong
☐ I cannot assess

Medication availability during quarantine (e.g., video consultation followed by delivery).
☐ not at all
☐ a little
☐ strong
☐ very strong
☐ I cannot assess

The option of receiving advice despite a long distance to the nearest pharmacy.
☐ not at all
☐ a little
☐ strong
☐ very strong
☐ I cannot assess

The option of receiving advice despite limited mobility (e.g., bedridden, wheelchair-bound, illnesses with restricted mobility).
☐ not at all
☐ a little
☐ strong
☐ very strong
☐ I cannot assess

It's easier to address my concerns or illness in a familiar environment (e.g., home).
☐ not at all
☐ a little
☐ strong
☐ very strong
☐ I cannot assess

Comprehensive advice based on my health data in digital form (e.g., drug interactions).
Avoiding being overheard by other customers.
☐ not at all
☐ a little
☐ strong
☐ very strong
☐ I cannot assess

☐ Other reasons (please state):

8. On average, how much consultation time would you require for a remote consultation?
☐ < 3 minutes
☐ 3-5 minutes
☐ 6-10 minutes
☐ 11-20 minutes
☐ 21-30 minutes
☐ > 30 minutes
☐ Other reasons (please state):

9. How do you rate the following statements regarding remote consultations?
Video consultations are a good alternative for certain complaints (e.g., for unpleasant topics).
☐ disagree
☐ tend to disagree agree
☐ tend to agree
☐ agree
☐ I cannot asses

Video consultations are a good complement to in-person consultations (e.g., for acute infections).
☐ disagree
☐ tend to disagree agree
☐ tend to agree
☐ agree
☐ I cannot asses

I feel more comfortable receiving advice about a medication at a local pharmacy.
☐ disagree
☐ tend to disagree agree
☐ tend to agree
☐ agree
☐ I cannot asses

I would feel more comfortable using a video consultation if I already knew the consultant or the local pharmacy.
☐ disagree
☐ tend to disagree agree
☐ tend to agree
☐ agree
☐ I cannot asses

I would like to use video consultations with local pharmacies in the future.
☐ disagree
☐ tend to disagree agree
☐ tend to agree
☐ agree
☐ I cannot asses

10. In your opinion, who should cover the costs for a remote consultation at your local community pharmacy?
☐ My health insurance
☐ The pharmacy
☐ Other:

11. In principle, would you be willing to cover the costs of a remote consultation yourself?
☐ Yes, I would be willing to cover the entire cost myself.
☐ I would cover at least part of the cost myself.
☐ No, I am not willing to cover the cost.

12. Here you can write down any thoughts about remote consultations that you feel were not covered in the survey.

Finally, we ask you to provide information about yourself and your use of technical devices for the evaluation of the survey.

13. Which of the following technical devices would you use for a remote consultation?
☐ Smartphone
☐ Tablet
☐ Laptop/Notebook/Computer
☐ Other devices:
☐ I cannot asses

14. How much time do you spend daily using your smartphone, tablet, and/or computer for personal use?
☐ <1 hour
☐ 3-4 hours
☐ 1-2 hours
☐ 5-6 hours
☐ 6 hours
☐ I don't spend any time on it at all.

15. How would you rate your digital skills in general?
☐ unsatisfactory
☐ poor
☐ sufficient
☐ satisfactory
☐ good
☐ very good
☐ I cannot asses

16. Do you have a regular pharmacy?
☐ yes
☐ no

17. On average, how often do you visit a pharmacy?
Please select the most appropriate answer.
☐ Daily
☐ Weekly
☐ Monthly
☐ Quarterly (every 3 months)
☐ Annually
☐ Less than annually

18. How many minutes do you need to reach the pharmacy you visit most frequently?
☐ < 5 minutes
☐ 5-15 minutes
☐ 16-30 minutes
☐ > 30 minutes
☐ I cannot asses

19. Do you have any of the following diseases?
☐ Multiple choices possible
☐ Cardiovascular diseases (e.g., high blood pressure, heart failure, cardiac arrhythmias)
☐ Disorders of lipid metabolism (e.g., high cholesterol)
☐ Metabolic diseases (e.g., type 1/type 2 diabetes mellitus, gout, thyroid diseases)
☐ Psychologically based diseases (e.g., anxiety, nervous restlessness, depression)
☐ Neurological diseases (e.g., multiple sclerosis, Alzheimer's disease, epilepsy,
☐ Parkinson's disease)
☐ Chronic pain (e.g., migraines, chronic headaches)
☐ Eye diseases (e.g., glaucoma, cataracts)
☐ Lung diseases (e.g., asthma, COPD)
☐ Allergies and intolerances (e.g., hay fever, lactose intolerance)
☐ Skin diseases (e.g., atopic dermatitis, psoriasis)
☐ Gastrointestinal diseases (e.g., ulcerative colitis, rheumatoid arthritis) Crohn's disease)
☐ Diseases of the urethra/bladder (e.g., prostate disease, bladder emptying disorders)
☐ Sex hormone imbalance (e.g., menopause, hormone-related cancers)
☐ Rheumatological diseases (e.g., rheumatoid arthritis)
☐ Other (please state):
☐ None of the above-mentioned diseases are present.
☐ No answer

20. On average, how many medications do you receive from a doctor per quarter? (1 quarter = 3 months)
☐ 0-1
☐ 2-3
☐ 4-5
☐ >5

21. On average, how many medications do you buy without a prescription from a pharmacy per quarter? (1 quarter = 3 months)
☐ 0-1
☐ 2-3
☐ 4-5
☐ >5

22. What gender do you consider yourself to be?
☐ Female
☐ Male
☐ Diverse

23. How old are you (in years)?
☐ <21
☐ 31-40
☐ 51-60
☐ 71-80
☐ 21-30
☐ 41-50
☐ 61-70
☐ 80

24. What is your postal code?
If you are not from Germany, please provide the license plate number for your country.

25. What is the size of the city or municipality where you live (measured by population)?
☐ Large city (over 100,000 inhabitants)
☐ Medium-sized city (over 20,000 inhabitants)
☐ Small town (over 5,000 inhabitants)
☐ Rural area (under 5,000 inhabitants)
☐ Not specified

26. What is your highest general education qualification?
☐ (Previously) no school leaving certificate
☐ Hauptschulabschluss (9th grade)
☐ Realschulabschluss (10th grade)
☐ Fachhochschulreife (university entrance qualification)
☐ Abitur (university entrance qualification)
☐ Another school leaving certificate, namely:

27. What vocational qualifications do you have?
Multiple selections possible
☐ (Previously) without a professional qualification
☐ Vocational training
☐ Bachelor's degree
☐ Master's degree, Magister degree, State Examination
☐ Diploma
☐ Doctorate
☐ Another professional qualification, namely:

Thank you for taking the time to answer the questions.

The team at Sanct Georg Pharmacies and the Clinical Pharmacy at the University of Leipzig would like to thank you very much for your help and support.
If we have piqued your interest in a remote consultation, please speak to the pharmacy staff or visit us on our website.
We would also be grateful if you would agree to participate in a follow-up survey after completing a remote consultation.
If you are interested or have further questions, the pharmacy team at Sanct Georg Pharmacies and the Clinical Pharmacy at the University of Leipzig will be happy to help.
Your answers have been saved; you can now close the browser window.

Nathalie Szafarczyk (PhD student in Clinical Pharmacy at the University of Leipzig)
Chris Graichen (Head of Pharmacy at the Sanct Georg Pharmacy)
Philipp Harand (Graduate student in Clinical Pharmacy at the University of Leipzig)
Sanct Georg Pharmacy, Delitzscher Straße 137, 04129 Leipzig
Sanct Georg Pharmacy, Delitzscher Landstraße 62, 04158 Leipzig
